# Controlled human malaria infection (CHMI) outcomes in Kenyan adults is associated with prior history of malaria exposure and anti-schizont antibody response

**DOI:** 10.1186/s12879-022-07044-8

**Published:** 2022-01-24

**Authors:** Melissa C. Kapulu, Domtila Kimani, Patricia Njuguna, Mainga Hamaluba, Edward Otieno, Rinter Kimathi, James Tuju, B. Kim Lee Sim, CHMI-SIKA Study Team, Abdirahman I. Abdi, Yonas Abebe, Philip Bejon, Peter F. Billingsley, Peter C. Bull, Zaydah de Laurent, Stephen L. Hoffman, Eric R. James, Silvia Kariuki, Sam Kinyanjui, Cheryl Kivisi, Johnstone Makale, Kevin Marsh, Khadija Said Mohammed, Moses Mosobo, Janet Musembi, Jennifer Musyoki, Michelle Muthui, Jedidah Mwacharo, Kennedy Mwai, Joyce M. Ngoi, Omar Ngoto, Irene Nkumama, Francis Ndungu, Dennis Odera, Bernhards Ogutu, Fredrick Olewe, Donwilliams Omuoyo, John Ong’echa, Faith Osier, Thomas L. Richie, Jimmy Shangala, Juliana Wambua, Thomas N. Williams

**Affiliations:** 1 Centre for Geographic Medicine Research (Coast), Kenya Medical Research Institute-Wellcome Trust Research Programme, P. O. Box 230, Kilifi, 80108 Kenya; 2 Centre for Tropical Medicine and Global Health, Nuffield Department of Medicine, University Oxford, Oxford, OX3 7LG UK; 3 Sanaria Inc., Rockville, MD 20850 USA

**Keywords:** Malaria exposure, Controlled human malaria infection, *Plasmodium falciparum*, Anti-schizont antibody response

## Abstract

**Background:**

Individuals living in endemic areas acquire immunity to malaria following repeated parasite exposure. We sought to assess the controlled human malaria infection (CHMI) model as a means of studying naturally acquired immunity in Kenyan adults with varying malaria exposure.

**Methods:**

We analysed data from 142 Kenyan adults from three locations representing distinct areas of malaria endemicity (Ahero, Kilifi North and Kilifi South) enrolled in a CHMI study with *Plasmodium falciparum* sporozoites NF54 strain (Sanaria® PfSPZ Challenge). To identify the in vivo outcomes that most closely reflected naturally acquired immunity, parameters based on qPCR measurements were compared with anti-schizont antibody levels and residence as proxy markers of naturally acquired immunity.

**Results:**

Time to endpoint correlated more closely with anti-schizont antibodies and location of residence than other parasite parameters such as growth rate or mean parasite density. Compared to observational field-based studies in children where 0.8% of the variability in malaria outcome was observed to be explained by anti-schizont antibodies, in the CHMI model the dichotomized anti-schizont antibodies explained 17% of the variability.

**Conclusions:**

The CHMI model is highly effective in studying markers of naturally acquired immunity to malaria.

*Trial registration* Clinicaltrials.gov number NCT02739763. Registered 15 April 2016

**Supplementary Information:**

The online version contains supplementary material available at 10.1186/s12879-022-07044-8.

## Background

*Plasmodium falciparum* malaria remains a pressing global health emergency. Encouraging progress in its control has been made in some areas of Africa [1], but elimination does not appear realistic in many areas. The current lead vaccine candidates are based on the circumsporozoite protein (CSP) and have been shown to be protect against clinical manifestations of *P. falciparum* disease in children [2, 3]. Higher vaccine efficacy against clinical manifestations might be achievable through inducing immune responses against antigens from the asexual blood-stages [4]. The clinical development pathway for any one candidate vaccine is expensive and lengthy. None of the blood-stage candidate vaccines subjected to field trials have progressed to Phase III trials [5, 6]. The need to understand and interrogate naturally acquired immunity to malaria is fundamental to antigen selection and vaccine design. A common approach is to use immuno-epidemiological studies in malaria endemic regions, where immunological responses from cross-sectional surveys of children are linked with the risk of subsequent malaria episodes [7–11]. A limitation of this approach has been the reliance on uncontrolled natural but heterogenous exposure to malaria [8, 9, 12] as well as exposure to genetically diverse parasites [13] in the field.

Controlled Human Malaria Infection (CHMI) studies have the potential to accelerate the selection of antigens for vaccine development by controlling for malaria exposure, including parasite strain, as well as level of infectious dose. For ethical reasons, CHMI requires adult volunteers rather than children. In endemic areas immunity is acquired with age and adults usually have high levels of immunity to the consequences of infection [14]. Nevertheless, even among adults’ levels of immunity may be variable. We have recently described clinical outcomes and safety of CHMI in Kenyan adults after infection with cryopreserved viable, aseptic, and purified *Plasmodium falciparum* sporozoites (PfSPZ Challenge) at a dose of 3200 injected by syringe [15]. We showed that using CHMI in this population of 142 pre-exposed adults, 26 (18.3%) had febrile symptoms and were treated; 30 (21.1%) reached ≥ 500 parasites/µl and were treated; 53 (37.3%) had parasitaemia without meeting thresholds for treatment and; whilst 33 (23.2%) remained qPCR negative (in a subset of volunteers, some of those qPCR negative between days 8 and 10 post-infection had low parasitaemia in comparison to two other qPCR methods) [15]. These findings are consistent with other CHMI studies in volunteers from endemic areas [16, 17]. However, the outcomes of CHMI that are most strongly associated with naturally acquired immunity have not yet been determined. Furthermore, categorizations into multi-level descriptive outcomes do not maximize analytical power for correlates of immunity, and either a binary classification or a continuous variable would be analytically optimal.

We therefore in this study conducted an analysis using anti-schizont antibody responses and location of residence as surrogates of immunity. We examined various parameters from the patterns of parasite growth during CHMI. This was to determine which parameters most closely associated with these two surrogates of immunity, and to identify whether any were more discriminatory of host immunity than the standard immuno-epidemiological studies conducted in the field.

## Methods

### Study design and population

The full protocol [18] and description of safety and outcome [15] has been published. Briefly, data from the CHMI-SIKA study, which was an open, un-blinded and non-randomised with all volunteers receiving an intravenous injection [direct venous inoculation (DVI)] dose of 3.2 × 10^3^ PfSPZ Challenge PfNF54 strain (i.e. cryopreserved, infectious sporozoites). The volunteers were monitored for blood parasitaemia by quantitative polymerase chain reaction (qPCR) to determine parasite growth. The 3.2 × 10^3^ PfSPZ dose was selected because this has infected 100% malaria-naïve volunteers undergoing CHMI in studies in the US and EU [19, 20]. PfNF54 is African in origin and it is therefore expected that < 100% of African volunteers with well-developed naturally acquired immunity will become infected [21].

### Anti-malarial drug concentration

We retrospectively measured the concentrations of anti-malarial drugs (artemether, dihydroartemisinin, sulfadoxine, pyrimethamine, chloroquine, lumefantrine, and desbutyl-lumefantrine), retrospectively at a day before challenge (C − 1) and after challenge (at C + 8) [15]. We excluded those with drug levels above the minimum inhibitory concentration (MIC) for lumefantrine but retained those with levels below the MIC for sulfadoxine (in absence of pyrimethamine) and with trace levels of chloroquine as described previously [15].

### Anti-schizont antibody levels

Plasma samples were tested by ELISA for the presence of human IgG against schizont extract as described previously [22, 23]. *P. falciparum* 3D7 strain parasites were cultured to schizont stage to make a preparation of schizont extract. To run the ELISAs, the extract was used to coat high absorbance plates at an established concentration shown to have saturation of responses using plasma from hyper immune individuals. The assay was repeated if duplicate optical density (OD) values for an individual plasma sample varied by more than a factor of 1.5. A pool of serum samples from an area in Africa where malaria is highly endemic was titrated on each plate and acted both as a positive control and provided values for a standard curve for converting optical density (OD) readings into concentrations (Antibody Units, AU).

### Location of residence

Volunteers were recruited from differing malaria endemic regions in Kenya: Ahero in Western Kenya (moderate to high transmission region); Kilifi North on the Kenyan Coast (low to no malaria transmission); and Kilifi South (moderate transmission region) [1, 24]. In this analysis volunteers from Ahero and Kilifi South were combined as resident at “high transmission” intensity and Kilifi North taken as resident at “low transmission” intensity.

### Parasite detection by qPCR

For parasite detection, venous blood samples were collected twice every day from days 8 to 15 of CHMI and then once every day from days 16 to 22 of CHMI for qPCR analysis by detection of the 18 S ribosomal RNA *P. falciparum* gene [15] in triplicates in a TaqMan assay using primers and probes previously described [25]. Non-template control was used as a negative control (in triplicate wells) with parasite quantification against known cultured parasite standards comprising of 6 serial dilutions of extracted DNA also run in triplicates. The cultured parasite standards were produced in 3 different batches. Selected samples were re-ran from each CHMI cohort against a final set of standards, including the WHO external quantified quality control sample [26].

### Statistical analysis

PCR results are presented as the geometric mean of three replicate assays at each timepoint. Time to treatment was the number of days between challenge and the treatment decision taken either because: (a) the volunteer reached the pre-assigned threshold of 500 *falciparum* parasites/ml by qPCR; (b) they had developed febrile symptoms and clinicians had treated them at lower parasite density; or (c) they reached the end of the study without reaching parasite density threshold or having symptoms. Other parameters to describe the outcomes were derived from qPCR results as follows: (i) the time to particular parasite density thresholds, where volunteers not reaching those thresholds were described as missing data; (ii) the “proportion of days growing” where any consecutive increase in parasite density is considered a “day growing” (this was calculated from raw data, then also from smoothed data taking the moving average over 2 days); (iii) the mean parasite density as a geometric mean, excluding timepoints after treatment; (iv) the maximum number of days of continuous consecutive growth; (v) the gradient of growth from a best-linear-fit of the period defined in (iv); (vi) the median number of days since challenge for the days of parasite growth as defined in (ii); (vii) the converse of (ii), (iv), and (v); (viii) for days of decline in parasite density rather than growth; (ix) the “inoculum” defined as the peak parasite density observed between days 8.5 and 10 after challenge and; (x) the “variability” calculated as the summed day-to-day variation in parasite density. Kruskal–Wallis tests with multiple comparisons were used to compare anti-schizont antibodies by location and qPCR outcome and Spearman’s rank correlation was used to explore correlations between anti-schizont antibody, location, and qPCR parameters.

Survival models were developed using Cox regression in three stages; (a) univariable analysis of all potential independent predictors; and (b) multivariable analysis including significant predictors from (a); second multivariable analysis retaining only significant predictors from (b). The variability in outcome explained by anti-schizont antibodies was calculated using pseudo r^2^. To compare the CHMI cohort with a previous observational field study of children [9, 11, 23], the antibody levels were divided into two groups (above and below the median), and analysis of the child cohort was restricted to the asymptomatically infected group where the protective effect of anti-schizont antibodies had been shown to be most evident [8, 11, 23].

## Results

### Anti-schizont antibody responses for the volunteers enrolled in the study

Data from 142 volunteers were included in the analysis as previously described [15]. The median age of the volunteers was 28 years old (range 18–45) and 30% were female. Antibody responses to schizont extract were measured for all the volunteers at screening (Additional file 1: Fig. S1). Volunteers from Kilifi North had significantly lower anti-schizont antibodies (median of 896 Antibody units (AU), 95% CI 566 to 1473) compared with volunteers from Kilifi South (median of 9238 AU, 95% CI 6399 to 12,324, *p *< 0.00001) and Ahero (median of 4666 AU, 95% CI 966 to 28,702, *p *< 0.00054) but volunteers from Kilifi South and Ahero had similar antibody levels (*p *= 0.085). For further analysis, volunteers from Kilifi North (N = 34) were considered to be residents of an area of “low transmission” whilst volunteers from Kilifi South (N = 93) were combined with Ahero volunteers (N = 15) and considered to be residents of an area of “high transmission”.

### qPCR categorized outcomes in relation to location and antibody response

We had previously observed four distinct outcomes based on parasite growth as measured by qPCR following CHMI [15] being parasite growth by qPCR meeting the threshold criteria for malaria diagnosis (≥ 500 parasites/ml) either: (a) with fever (i.e. “treated febrile”); (b) without fever but reaching a parasite density requiring treatment (i.e. “treated non-febrile”); (c) with parasites detected by qPCR but not at a parasite density meeting the threshold criteria for treatment (i.e. “PCR positive untreated”); or (d) parasites not identified by qPCR throughout monitoring (i.e. “PCR negative”) (Additional file 2: Fig. S2). Volunteers who were “treated febrile” had lowest anti-schizont antibodies and were least likely to be residents of the high transmission areas (Table 1, Additional file 3: Fig. S3). The “treated non-febrile” group had intermediate levels of anti-schizont antibodies and intermediate likelihood of being residents of the high transmission area. The “untreated PCR positive” group and then “PCR negative” group had high levels of anti-schizont antibodies and were both very likely to be residents of the high transmission areas. Those who were qPCR positive untreated could further be examined in sub-groups by dividing them into those who were positive either early, late, or throughout the period of qPCR monitoring. We did not identify any significant differences in anti-schizont antibodies or location of residence for these additional sub-groups (Additional file 5: Table S1).

**Table 1:** qPCR outcome in relation to anti-schizont antibody responses

Outcome	N	Anti-schizont antibody concentration (AU)^a^	Proportion resident in high transmission areas^b^
Treated febrile	26	794 (501 to 1230)	0.31 (0.12 to 0.49)
Treated non-febrile	30	3311 (1585 to 7080)	0.56 (0.38 to 0.75)
Untreated PCR (+)	53	8710 (6166 to 12,589)	0.98 (0.93 to 1.0)
PCR (−)	33	15,849 (8913 to 31,623)	0.93 (0.86 to 1.0)

PCR (+) and PCR (−) refer to volunteers who were PCR positive and negative respectively; N is the total number of volunteers in each outcome category

^a^Median antibody responses with 95% CI in parenthesis

^b^Proportion with 95% CI in parenthesis. N number of volunteers in the analysis

### qPCR parameter associations with location and antibody response

We examined various parameters that described the qPCR results per individual volunteer (Table 2). The strongest non-parametric correlates of location of residence (i.e. residence at high vs. low transmission intensity) or anti-schizont antibodies were time to reaching a threshold of 250 parasites/ml; time to treatment; and the categorization of treatment versus no treatment (Table 2). Other parameters that were strong correlates of location of residence or of anti-schizont antibodies were highly cross correlated with each other (Additional file 4: Fig. S4) and we did not identify a second independent predictor using parametric analyses after adjusting for time to treatment (Additional file 5: Table S2). The parameter time to treatment was used for further analysis over the use of time to a threshold of 250 parasites/ml since some volunteers were treated at lower parasite densities leading to missing data for time to 250 parasites/ml.

**Table 2:** Non-parametric analysis of qPCR parameters with anti-schizont antibody responses and location

Parameter	Rho anti-schizont antibody	*p* value anti-schizont antibody	Rho location	*p* value location	N
Inoculum^a^	− 0.25	**0.003**	− 0.08	0.32	142
Time to treatment	0.56	**3.60e−13**	0.64	**1.85e−17**	142
Treated vs. untreated	− 0.54	**2.47e−12**	− 0.59	**6.59e−15**	142
Mean parasite density	− 0.53	**1.26e−11**	− 0.44	**6.07e−08**	142
Proportion of days with parasite growth^b^	− 0.54	**3.40e−12**	− 0.43	**7.34e−08**	142
Proportion of days with parasite growth^c^	− 0.52	**2.93e−11**	− 0.41	**4.18e−07**	142
Proportion of days with declining parasite numbers	− 0.34	**0.00004**	− 0.26	**0.002**	142
Days of longest consecutive parasite growth	− 0.5	**1.63e−10**	− 0.39	**1.00e−06**	142
Median point of days with parasite growth	0.26	**0.01**	0.39	**0.00008**	96
Maximum days of consecutive decline	− 0.12	0.24	− 0.01	0.88	104
Median day of decline	0.02	0.82	0.05	0.61	104
Gradient	− 0.45	**2.15e−08**	− 0.47	**3.47e−09**	142
Variability^d^	0.19	0.06	0.06	0.53	96
Time to threshold of parasites (1/µl)	0.33	**0.00006**	0.27	**0.001**	142
Time to threshold of parasites (5/µl)	0.45	**2.83e−08**	0.49	**4.82e−10**	142
Time to threshold of parasites (50/µl)	0.54	**3.18e−12**	0.6	**2.36e−15**	142
Time to threshold of parasites (250/µl)	0.62	**9.34e−14**	0.62	**9.69e−14**	118
Time to threshold of parasites (500/µl)	0.54	**1.37e−09**	0.56	**1.71e−10**	111
Time to threshold of parasites (1000/µl)	0.51	**1.33e−08**	0.59	**1.28e−11**	108

N number of volunteers in the analysis. Analysis uses Spearman’s rank-order correlation.* P* values in bold indicate statistical significance (*p* < 0.05)

^a^Peak at days considered are from days 8.5 to 10 post-infection

^b^Analysis of smoothed data

^c^Analysis of raw data

^d^Represents the summed/average day to day increase or decrease

### Survival analysis

**Fig. 1:**
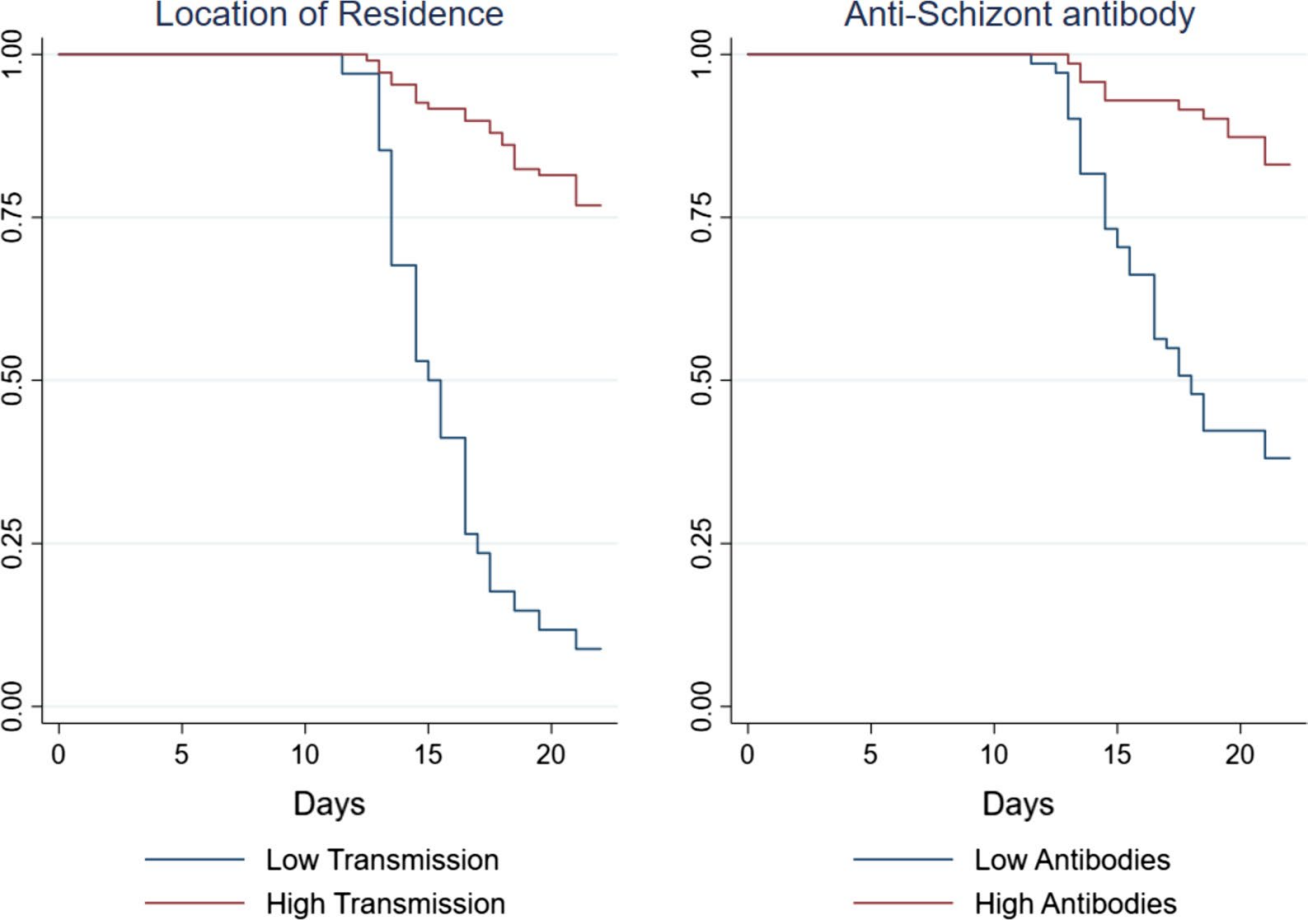
Time to treatment survival analysis. Kaplan–Meier curves comparing time to treatment with location of residence (left panel) and anti-schizont antibody response (right panel). Shown are survival curves for location of residence is low transmission (blue) vs. high transmission (red). For anti-schizont antibody responses shown is low antibodies (blue) vs. high antibodies (red)

We developed a multivariable Cox regression model of time to treatment, finding both anti-schizont antibodies and location of residence to be strong independent predictors of outcome (Table 3). The presence of parasites at screening and plasma lumefantrine drug concentrations were weak predictors of outcome in univariable analysis (Table 3), but not in multivariable analysis (Multivariable 1, Table 3). Parasites at screening and lumefantrine drug concentrations were both confounded by location of residence (r = 0.20, *p *= 0.016 and r = 0.30, *p *= 0.0003 for associations with location of residence, respectively). The year of enrolment in the trial (cohort year), anti-malarial drug concentration, age, and gender were not significant predictors of outcome. In the final model (Multivariable 2), the two independent predictors were residence (i.e. at high vs. low transmission) and anti-schizont antibody concentration, explaining 35% of the variability in outcome on logistic regression (Table 3, and Fig. 1).

**Table 3:** Cox regression analysis of time to treatment

Variable	Univariable			Multivariable 1			Multivariable 2		
	HR	95% CI	*p* value	HR	95% CI	*p*	HR	95% CI	*p*
Cohort (i.e. one cohort per year)									
2016	1								
2017	0.74	0.42, 1.32	0.31						
2018	0.87	0.51, 1.50	0.62						
Age (years)	1	0.97, 1.04	0.82						
Residence at low transmission^a^	1			1			1		
Residence at high transmission^a^	0.11	0.06, 0.19	**1 × 10**^**−14**^	0.22	0.11, 0 0.44	**0.00003**	0.20	0.10, 0.40	**5 × 10**^**−6**^
Sulfadoxine^b^	1.21	0.78, 1.88	0.4						
Lumefantrine^b^	0.55	0.32, 0.96	**0.04**	0.78	0.45, 1.34	0.37			
Anti-Schizont	0.23	0.14, 0.36	**2 × 10**^**−10**^	0.51	0.30, 0.88	**0.02**	0.44	0.27, 0.74	**0.0016**
qPCR (+) at screening	0.17	0.05, 0.55	**0.003**	0.39	0.11, 1.36	0.14			
Sex (male)	1.1	0.62, 1.94	0.75						

PCR (+) volunteers, qPCR positive at screening.* P* values in bold indicate statistical significance (*p* < 0.05)

^a^Residence of volunteers

^b^Log transformed concentration values used

**Fig. 2:**
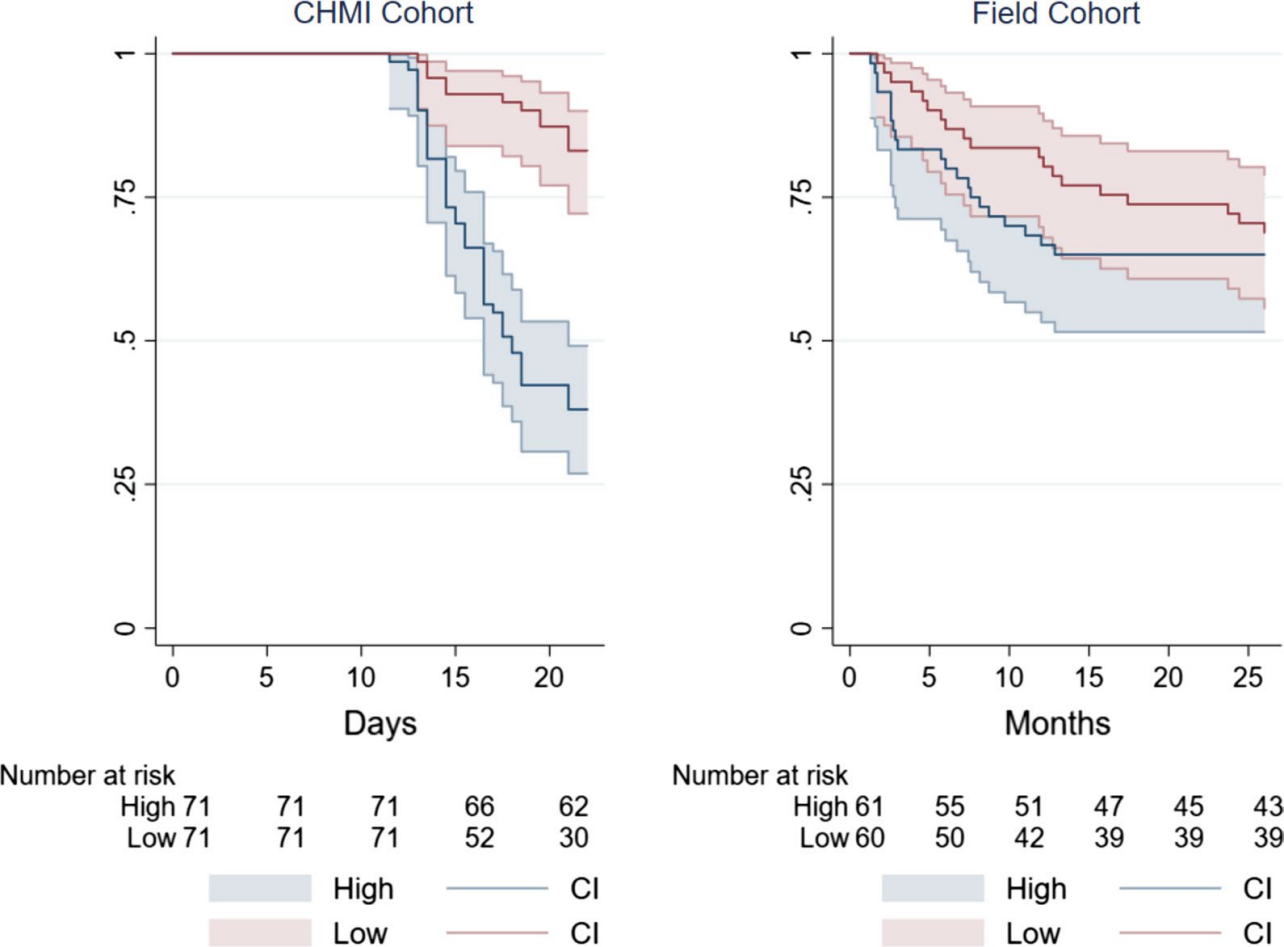
Survival analysis of CHMI vs. field-based observational study. Kaplan–Meier curves comparing of the CHMI cohort (left panel) and field-based cohort (right panel) in relation to requirement for treatment and anti-schizont antibody responses. Antibody responses are shown as low (red) vs. high (blue)

We compared the predictive strength of anti-schizont antibodies in the CHMI model with previous cohort studies based on natural exposure in the field [9, 11, 23], to determine whether the CHMI model would advance the field in examining for correlates of infection. In order to make comparisons across models, we used dichotomized anti-schizont antibody levels above and below the median for each study to have a bi-level comparison in each setting that was not dependent on the different range of antibody levels. We compared the pseudo R^2^ in logistic regression to determine the variability in outcome explained by antibody levels in each setting. In CHMI, the odds ratio (OR) of requiring treatment based on anti-schizont antibody levels above the median was OR = 0.12 (95% CI 0.06 to 0.27, p = 2 **× **10^−7^) and explained 17% of the variability. In the previously reported cohort of 121 children between the ages of 1 and 8 years found to be parasite positive at baseline with the inclusion criteria for analysis being residence in the study area, having anti-schizont antibodies above the median level was associated with OR = 0.64 (95% CI 0.29 to 1.4, *p *= 0.26) for febrile malaria, explaining 0.8% of the variability in outcome. Survival plots from the CHMI study showed a clear distinction in time to treatment by anti-schizont antibody responses (Fig. 2, left panel), in contrast to the less clear distinction seen in field studies based on natural exposure (Fig. 2, right panel).

## Discussion

We used serial qPCR to determine the outcomes most strongly associated with anti-schizont antibodies and location of residence (low vs. high transmission), in order to define the outcomes for CHMI in exposed adults that are most strongly associated with surrogates of immunity. We used anti-schizont antibody levels and location of residence at varying prior exposure to malaria as surrogates for immunity to malaria. We examined several potential parameters based on the qPCR monitoring done for CHMI for their association with anti-schizont antibodies and location. Time to treatment and time to 250 parasites/µl were strongly associated with anti-schizont antibodies and with location. We preferred time to treatment rather than time to 250 parasites/µl, as the former included the full set of volunteer data and avoids the potential bias of missing data from volunteers who were treated before reaching 250 parasites/µl.

After adjusting for time to treatment, there were no other independent predictors of anti-schizont antibodies or of location of residence. We therefore developed a survival analysis based on time to treatment. The combination of location of residence and anti-schizont antibodies as a continuous variable explained 35% of the variability in time to treatment in CHMI. Since prior residence and anti-schizont antibodies only offer limited information on the true extent of host immunity, this implies that a very significant proportion of the variability in outcome in CHMI is due to host immunity.

We examined whether the analysis of CHMI for naturally acquired immunity was a significant advance over previous studies conducted in the field based on natural exposure to malaria. Adults have higher levels of immunity than children, different endpoints are used for adults participating in CHMI compared with children in field observational studies, but nevertheless these study designs both share the aim to define potential correlates of immunity. In order to make comparisons, we used logistic regression with febrile malaria as the outcome in the field studies, and with treatment criteria as the outcome in CHMI. We used anti-schizont antibodies as the predictor variable. Levels of anti-schizont antibodies higher among adults than children, so we divided antibodies into high or low categories based on the median antibody level in each study. In the field-based observational study analysed here in a cohort of children, anti-schizont antibody responses explained less than 1% of the observed variability, but anti-schizont antibodies explained 17% of the variability in CHMI outcomes. This is not surprising given the variability in exposure to malaria seen in the field [8, 9], whereas in CHMI exposure is controlled and does not vary between participants.

This analysis, here, shows how, adjusting and accounting for heterogeneity of exposure and infection, and given that anti-schizont antibodies in field-based studies account for a small fraction of the variability, CHMI in an adult pre-exposed population has a larger discriminatory power to study immunity in relation to past exposure. Furthermore, in non-immune CHMI studies, a large proportion of the volunteers develop illness and require treatment at relatively low parasitaemia thresholds (between 5 and 50 parasites/ml) whilst in our study, individuals were often asymptomatic and parasite-free and this could largely be as a result of differences in responses in non-immunes with semi-immunes [27, 28]. With the exception of innate factors such as sickle cell trait [17], this resistance in previously malaria exposed individuals thus might be as a result of acquired adaptive immunity which is confirmed by anti-schizont antibody responses.

Thus, these findings presented here, provide a unique opportunity to advance the field of vaccine antigen discovery utilizing the CHMI platform with characterization and better understanding of the development of immunity to infection in the context of past malaria exposure. A comprehensive analysis of signatures or correlates of immunity as has been recent detailed utilizing systems serology approaches (both qualitative and quantitative antibody-based approaches) [29] will significantly advance the field.

This analysis, not relying on one or two parameters of the outcome measure (PCR) especially in the context of undertaking these studies in populations with varying past exposures to malaria is warranted. Traditionally, studies have relied on the parasite growth kinetics/rate as an important measure of outcome in CHMI studies—including as an assessment of vaccine or drug efficacy [30]. CHMI studies enrolling volunteers with a range of parasite exposures, to date in Africa, have taken the approach of endpoint measurement largely based on thick-blood microscopy at a particular threshold for diagnosis [17, 22, 31] to explain parasite growth rates. Achan et al. [16] despite using PCR as a criteria for endpoint, did not have the same breadth of past exposures as presented here. Hence, it is important for studies particularly utilizing PCR to undertake a detailed analysis of the most reliable parameter that would account for diversity in parasite growth.

## Conclusions

We thus conclude that CHMI studies in malaria endemic areas, using a standardized inoculum, are an effective platform and powerful tool with which to study host immunity. Variability in outcomes are more closely attributable to host immunity than for field-based studies based on natural exposure. Anti-schizont antibodies are generally not considered to be mechanistically related to immunity but rather a marker of past exposure. Anti-schizont antibodies are thus likely to be cross correlated with multiple other potential mechanisms of immunity [22, 23, 32, 33]. Hence in further studies of host immunity, we would expect antigen-specific responses and functional antibodies to explain remaining variability in outcome. It is possible that a few mechanistic markers will be independently associated with outcome and that, on adjusting, the associations with other markers of exposure will be attenuated, or that outcome will be explained by a range of parameters independently. In either case, the experimentally controlled conditions are expected to leave less unexplained variation than occurs in the field due to variable exposure to mosquito bites. In follow up analyses, immunological parameters, including but not limited to for example functional assays for blood-stage immunity [34, 35] and protein microarray analyses to identify blood-stage antigens [36], will need to be undertaken in relation to the PCR parameters described here. This will aid in the further identification of signatures or correlates of immunity. Previous immuno-epidemiological studies using observational cohorts have identified several immunological markers much more strongly associated with immunity than anti-schizont antibody [11], and these findings can now be tested using the CHMI approach in malaria endemic areas.

## Supplementary Information

**Additional file 1: Figure S1**. Schizont antibody responses by volunteer location. Violin plots of the anti-schizont antibody units (AU) measured from each of the 142 volunteers at screening by ELISA. Plasma samples obtained at screening from each individual volunteer were assessed forIgG specific responses to schizont extract. Indicated within each violin plot are boxplots with the median (dark solid line), minimum and maximum. Each individual is represented by each individual black closed circle.

**Additional file 2: Figure S2.** Parasite growth based on qPCR outcome and location. Blood samples from C + 8 onwards after inoculation were assessed by qPCR to determine parasitaemia from individuals from low transmission (left panel, Kilifi North—N = 34) and high transmission (right panel, Kilifi South—N = 93 and Ahero—N = 15). Parasitaemia was determined by asexual 18S ribosomal RNA gene qPCR done in Kilifi. Blue line s represent individuals who required treatment and reached diagnosis threshold (Treated); green lines represent individuals who did not meet the diagnosis threshold but were qPCR positive (Untreated PCR +ve); orange lines representindividuals who were qPCR negative throughout monitoring (PCR −ve); and red dot denotes individuals who were febrile and who required treatment and reached diagnosis threshold (Febrile).

**Additional file 3: Figure S3.** Anti-schizont antibody titres in relation to CHMI qPCR category outcomes. Violin plots of the anti-schizont antibody units (AU) measured from each of the 142 volunteers at screening by ELISA. Plasma samples obtained at screening from each individual volunteer were assessed for IgG specific responses to schizont extract. Indicated within each violin plot are boxplots with the median (dark solid line), minimum and maximum. Each individual is represented by each individual closed circle based on either low transmission (blue) or high transmission (peach).

**Additional file 4: Figure S4.** Spearman correlations of qPCR metrics. Correlation matrix of qPCR metrics showed collinearity for phenotype (treated vs untreated), time to thresholds (1000 parasites/ml; 500 parasites/ml; 250 parasites/ml; 50 parasites/ml; 5 parasites/ml; and 1 parasite/ml) or to diagnosis, and mean qPCR/gradient/days of growing (maximum days consecutively growing; median days of growth; proportion of days growing) or decline (maximum days consecutively decline; median days of decline; proportion of days declining), and other parasite growth metrics. Inoculum represents peak at days from days 8.5 to 10 post-infection; proportion of days with parasite growth represents analysis of smoothed data; proportion of days with parasite growth represents analysis of raw data; and variability represents the summed/average day to day increase or decrease.

**Additional file 5: Table S1.** Sub-group analysis of qPCR outcome in relation to anti-schizont antibody responses. **Table S2.** Parametric analysis of qPCR parameters with anti-schizont antibody responses and location.

## Data Availability

Data will be made available including data dictionaries after de-identification of volunteers. The data will be available to researchers who submit requests to dgc@kemri-wellcome.org to gain access to the data following a signed data access agreement. The study protocol, informed consent forms, and all other associated documents have been previously published.

## Abbreviations

AU: Antibody unit
CHMI: Controlled human malaria infection
CHMI-SIKA: Controlled human malaria infection in semi-immune Kenyan adults
DVI: Direct venous inoculation
ELISA: Enzyme linked immunosorbent assay
qPCR: Quantitative polymerase chain reaction
PfSPZ challenge: Aseptic, purified, cryopreserved *P. falciparum* sporozoites

## References

[1] Kamau A, Mogeni P, Okiro EA, Snow RW, Bejon P (2020). A systematic review of changing malaria disease burden in sub-Saharan Africa since 2000: comparing model predictions and empirical observations. BMC Med.

[2] Agnandji ST, Lell B, Soulanoudjingar SS, Fernandes JF, Abossolo BP, Conzelmann C (2011). First results of phase 3 trial of RTS,S/AS01 malaria vaccine in African children. N Engl J Med.

[3] Datoo MS, Natama MH, Somé A, Traoré O, Rouamba T, Bellamy D (2021). Efficacy of a low-dose candidate malaria vaccine, R21 in adjuvant Matrix-M, with seasonal administration to children in Burkina Faso: a randomised controlled trial. Lancet.

[4] Duffy PE, Patrick Gorres J (2020). Malaria vaccines since 2000: progress, priorities, products. npj Vaccines.

[5] Ogutu BR, Apollo OJ, McKinney D, Okoth W, Siangla J, Dubovsky F (2009). Blood stage malaria vaccine eliciting high antigen-specific antibody concentrations confers no protection to young children in Western Kenya. PLoS ONE.

[6] Thera MA, Doumbo OK, Coulibaly D, Laurens MB, Ouattara A, Kone AK (2011). A field trial to assess a blood-stage malaria vaccine. N Engl J Med.

[7] Fowkes FJI, Richards JS, Simpson JA, Beeson JG (2010). The relationship between anti-merozoite antibodies and incidence of *Plasmodium falciparum* malaria: a systematic review and meta-analysis. PLoS Med.

[8] Ndungu FM, Marsh K, Fegan G, Wambua J, Nyangweso G, Ogada E (2015). Identifying children with excess malaria episodes after adjusting for variation in exposure: identification from a longitudinal study using statistical count models. BMC Med.

[9] Bejon P, Warimwe G, Mackintosh CL, Mackinnon MJ, Kinyanjui SM, Musyoki JN (2009). Analysis of immunity to febrile malaria in children that distinguishes immunity from lack of exposure. Infect Immun.

[10] Beeson JG, Osier FH, Engwerda CR (2008). Recent insights into humoral and cellular immune responses against malaria. Trends Parasitol.

[11] Osier FH, Mackinnon MJ, Crosnier C, Fegan G, Kamuyu G, Wanaguru M (2014). Malaria: new antigens for a multicomponent blood-stage malaria vaccine. Sci Transl Med.

[12] Drakeley CJ, Corran PH, Coleman PG, Tongren JE, McDonald SLR, Carneiro I (2005). Estimating medium- and long-term trends in malaria transmission by using serological markers of malaria exposure. Proc Natl Acad Sci USA.

[13] Manske M, Miotto O, Campino S, Auburn S, Almagro-Garcia J, Maslen G (2012). Analysis of *Plasmodium falciparum* diversity in natural infections by deep sequencing. Nature.

[14] Kamau A, Mtanje G, Mataza C, Mwambingu G, Mturi N, Mohammed S (2020). Malaria infection, disease and mortality among children and adults on the coast of Kenya. Malar J.

[15] Kapulu MC, Njuguna P, Hamaluba M, Kimani D, Ngoi JM, Musembi J (2021). Safety and PCR monitoring in 161 semi-immune Kenyan adults following controlled human malaria infection. JCI Insight.

[16] Achan J, Reuling I, Yap XZ, Dabira E, Ahmad A, Cox M (2019). Serologic markers of previous malaria exposure and functional antibodies inhibiting parasite growth are associated with parasite kinetics following a *Plasmodium falciparum* controlled human infection. Clin Infect Dis.

[17] Lell B, Mordmuller B, Dejon Agobe JC, Honkpehedji J, Zinsou J, Mengue JB (2017). Impact of sickle cell trait and naturally acquired immunity on uncomplicated malaria after controlled human malaria infection in adults in Gabon. Am J Trop Med Hyg.

[18] Kapulu MCMC, Njuguna P, Hamaluba MMM, Abdi AIAI, Abebe Y, Audi A (2019). Controlled human malaria infection in semi-immune Kenyan adults (Chmi-sika): a study protocol to investigate in vivo plasmodium falciparum malaria parasite growth in the context of pre-existing immunity [version 2; peer review: 2 approved]. Wellcome Open Res.

[19] Gómez-Pérez GP, Legarda A, Muñoz J, Sim BKL, Ballester MR, Dobaño C (2015). Controlled human malaria infection by intramuscular and direct venous inoculation of cryopreserved *Plasmodium falciparum* sporozoites in malaria-naïve volunteers: effect of injection volume and dose on infectivity rates. Malar J.

[20] Mordmuller B, Supan C, Sim KL, Gomez-Perez GP, Ospina Salazar CL, Held J (2015). Direct venous inoculation of *Plasmodium falciparum* sporozoites for controlled human malaria infection: a dose-finding trial in two centres. Malar J.

[21] Moser KA, Drábek EF, Dwivedi A, Stucke EM, Crabtree J, Dara A (2020). Strains used in whole organism *Plasmodium falciparum* vaccine trials differ in genome structure, sequence, and immunogenic potential. Genome Med.

[22] Hodgson SH, Juma E, Salim A, Magiri C, Kimani D, Njenga D (2014). Evaluating controlled human malaria infection in Kenyan adults with varying degrees of prior exposure to *Plasmodium falciparum* using sporozoites administered by intramuscular injection. Front Microbiol.

[23] Osier FH, Fegan G, Polley SD, Murungi L, Verra F, Tetteh KK (2008). Breadth and magnitude of antibody responses to multiple *Plasmodium falciparum* merozoite antigens are associated with protection from clinical malaria. Infect Immun.

[24] Snow RW, Sartorius B, Kyalo D, Maina J, Amratia P, Mundia CW (2017). The prevalence of *Plasmodium falciparum* in sub-Saharan Africa since 1900. Nature.

[25] Ogwang C, Afolabi M, Kimani D, Jagne YJ, Sheehy SH, Bliss CM (2013). Safety and immunogenicity of heterologous prime-boost immunisation with *Plasmodium falciparum* malaria candidate vaccines, ChAd63 ME-TRAP and MVA ME-TRAP, in healthy Gambian and Kenyan adults. PLoS ONE.

[26] Cunningham JA, Thomson RM, Murphy SC, De La Paz Ade M, Ding XC, Incardona S (2020). WHO malaria nucleic acid amplification test external quality assessment scheme: results of distribution programmes one to three. Malar J.

[27] Church LWP, Le TP, Bryan JP, Gordon DM, Edelman R, Fries L (1997). Clinical manifestations of *Plasmodium falciparum* malaria experimentally induced by mosquito challenge. J Infect Dis.

[28] Epstein JE, Rao S, Williams F, Freilich D, Luke T, Sedegah M (2007). Safety and clinical outcome of experimental challenge of human volunteers with *Plasmodium falciparum*-infected mosquitoes: an update. J Infect Dis.

[29] Minassian AM, Silk SE, Barrett JR, Nielsen CM, Miura K, Diouf A (2021). Reduced blood-stage malaria growth and immune correlates in humans following RH5 vaccination. Med.

[30] Wockner LF, Hoffmann I, Webb L, Mordmüller B, Murphy SC, Kublin JG (2020). Growth rate of *Plasmodium falciparum*: analysis of parasite growth data from malaria volunteer infection studies. J Infect Dis.

[31] Shekalaghe S, Rutaihwa M, Billingsley PF, Chemba M, Daubenberger CA, James ER (2014). Controlled human malaria infection of Tanzanians by intradermal injection of aseptic, purified, cryopreserved *Plasmodium falciparum* sporozoites. Am J Trop Med Hyg.

[32] Hodgson SH, Llewellyn D, Silk SE, Milne KH, Elias SC, Miura K (2016). Changes in serological immunology measures in UK and Kenyan adults post-controlled human malaria infection. Front Microbiol.

[33] Murungi LM, Sondén K, Llewellyn D, Rono J, Guleid F, Williams AR (2016). Targets and mechanisms associated with protection from severe *Plasmodium falciparum* malaria in Kenyan children. Infect Immun.

[34] Joos C, Marrama L, Polson HEJ, Corre S, Diatta A-M, Diouf B (2010). Clinical protection from Falciparum malaria correlates with neutrophil respiratory bursts induced by merozoites opsonized with human serum antibodies. PLoS ONE.

[35] Osier FH, Feng G, Boyle MJ, Langer C, Zhou J, Richards JS (2014). Opsonic phagocytosis of *Plasmodium falciparum* merozoites: mechanism in human immunity and a correlate of protection against malaria. BMC Med.

[36] Kamuyu G, Tuju J, Kimathi R, Mwai K, Mburu J, Kibinge N (2018). KILchip v1.0: a novel *Plasmodium falciparum* merozoite protein microarray to facilitate malaria vaccine candidate prioritization. Front Immunol.

