# Supplementary figures and images for: Controlled human malaria infection (CHMI) outcomes in Kenyan adults is associated with prior history of malaria exposure and anti-schizont antibody response
Source: BMC Infect Dis. 2022 Jan 24;22:86. doi: 10.1186/s12879-022-07044-8 (PMC8785382; doi:10.1186/s12879-022-07044-8)

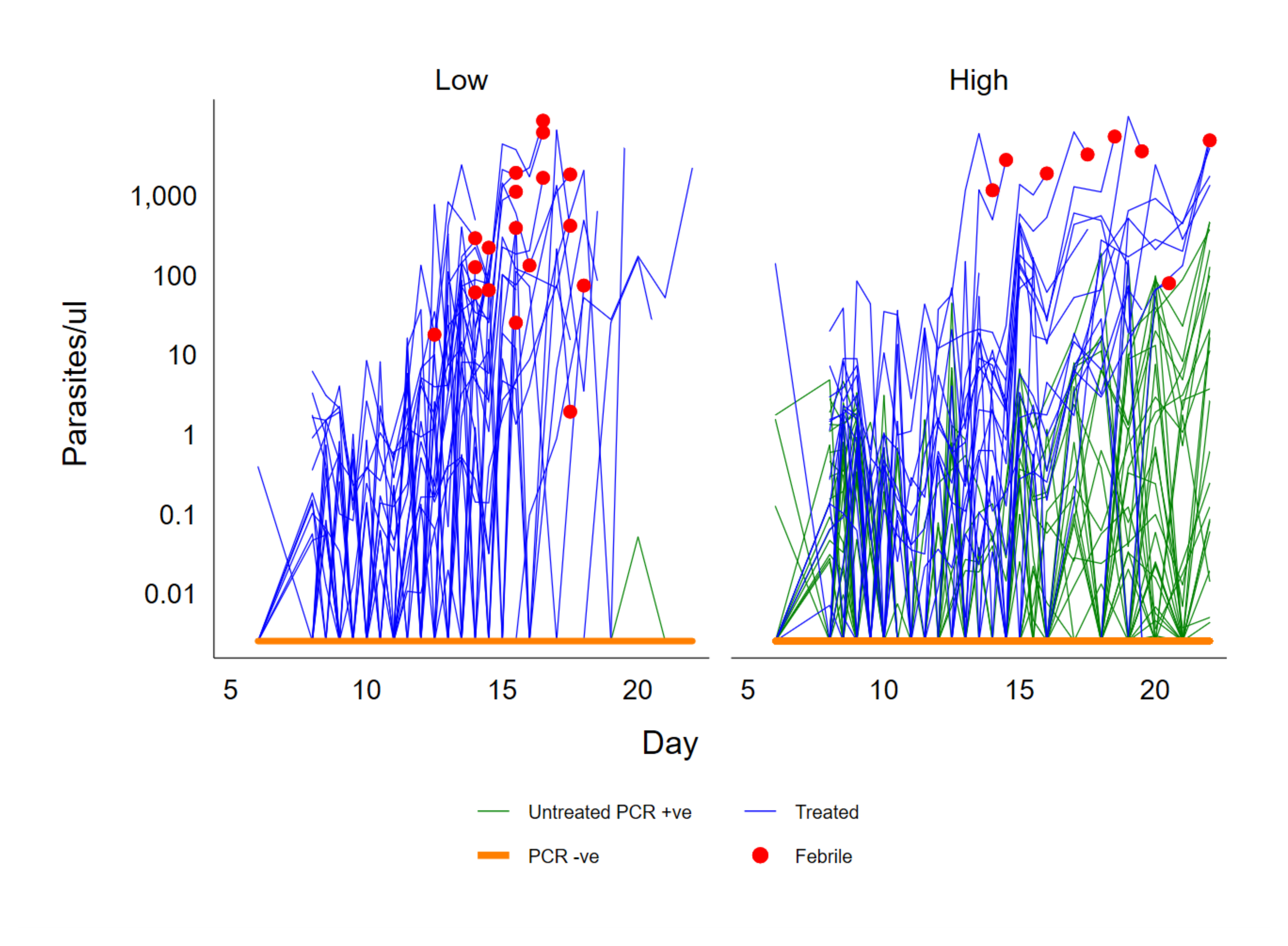

Supplement: Supplementary file 1 — Additional file 1: Figure S1. Schizont antibody responses by volunteer location. Violin plots of the anti-schizont antibody units (AU) measured from each of the 142 volunteers at screening by ELISA. Plasma samples obtained at screening from each individual volunteer were assessed forIgG specific responses to schizont extract. Indicated within each violin plot are boxplots with the median (dark solid line), minimum and maximum. Each individual is represented by each individual black closed circle. [file 12879_2022_7044_MOESM1_ESM.tif]

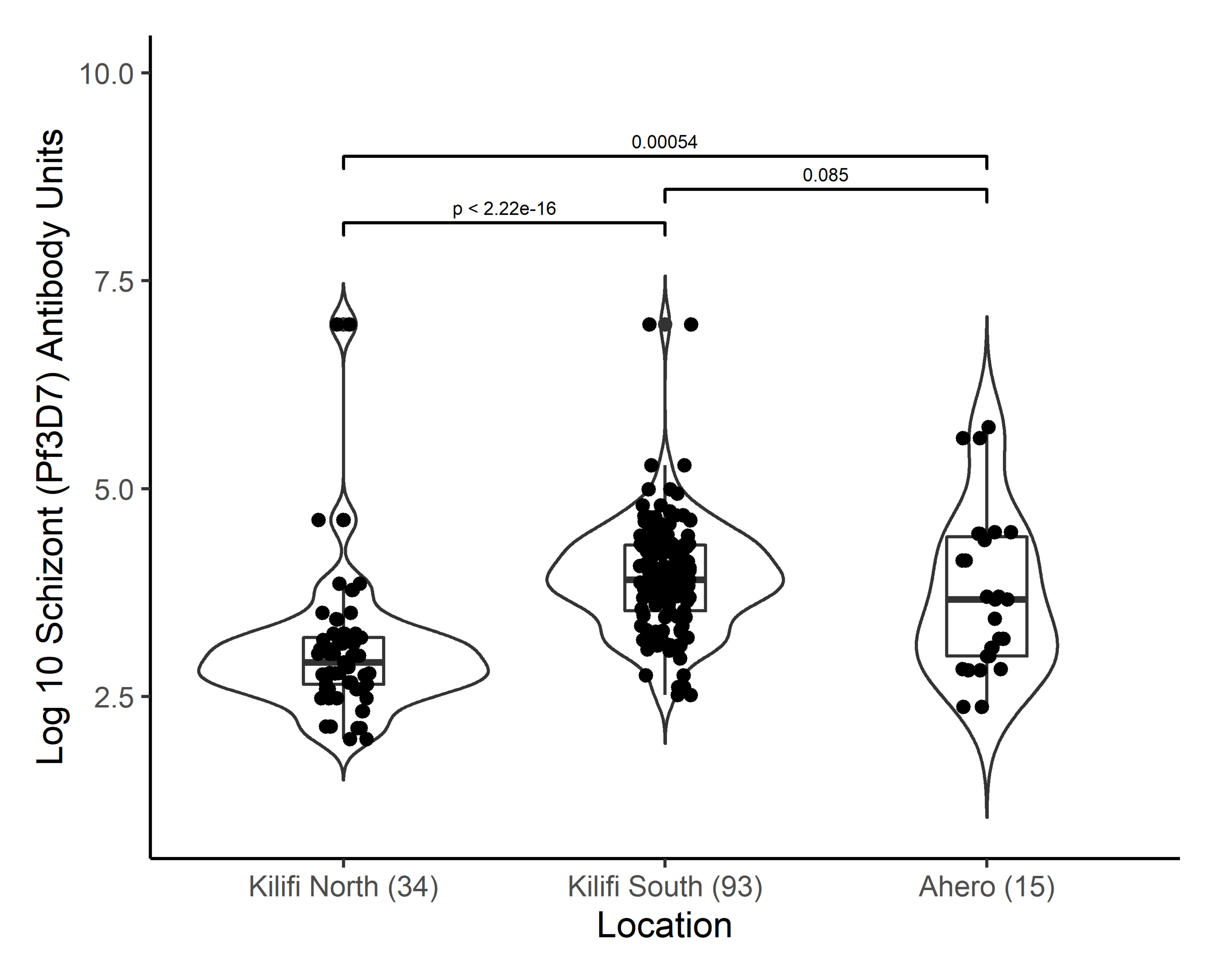

Supplement: Supplementary file 2 — Additional file 2: Figure S2. Parasite growth based on qPCR outcome and location. Blood samples from C + 8 onwards after inoculation were assessed by qPCR to determine parasitaemia from individuals from low transmission (left panel, Kilifi North—N = 34) and high transmission (right panel, Kilifi South—N = 93 and Ahero—N = 15). Parasitaemia was determined by asexual 18S ribosomal RNA gene qPCR done in Kilifi. Blue line s represent individuals who required treatment and reached diagnosis threshold (Treated); green lines represent individuals who did not meet the diagnosis threshold but were qPCR positive (Untreated PCR +ve); orange lines representindividuals who were qPCR negative throughout monitoring (PCR −ve); and red dot denotes individuals who were febrile and who required treatment and reached diagnosis threshold (Febrile). [file 12879_2022_7044_MOESM2_ESM.tif]

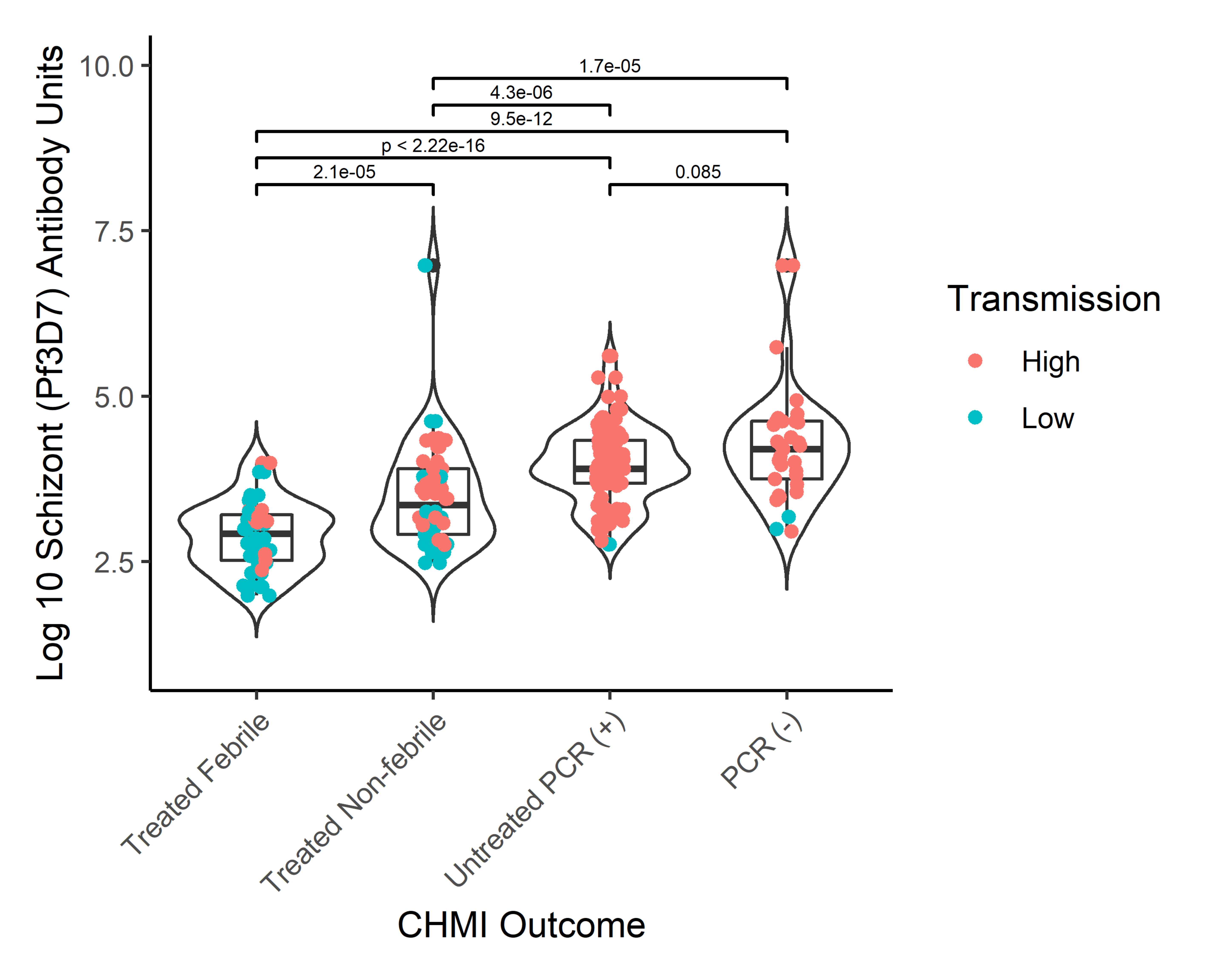

Supplement: Supplementary file 3 — Additional file 3: Figure S3. Anti-schizont antibody titres in relation to CHMI qPCR category outcomes. Violin plots of the anti-schizont antibody units (AU) measured from each of the 142 volunteers at screening by ELISA. Plasma samples obtained at screening from each individual volunteer were assessed for IgG specific responses to schizont extract. Indicated within each violin plot are boxplots with the median (dark solid line), minimum and maximum. Each individual is represented by each individual closed circle based on either low transmission (blue) or high transmission (peach). [file 12879_2022_7044_MOESM3_ESM.tif]

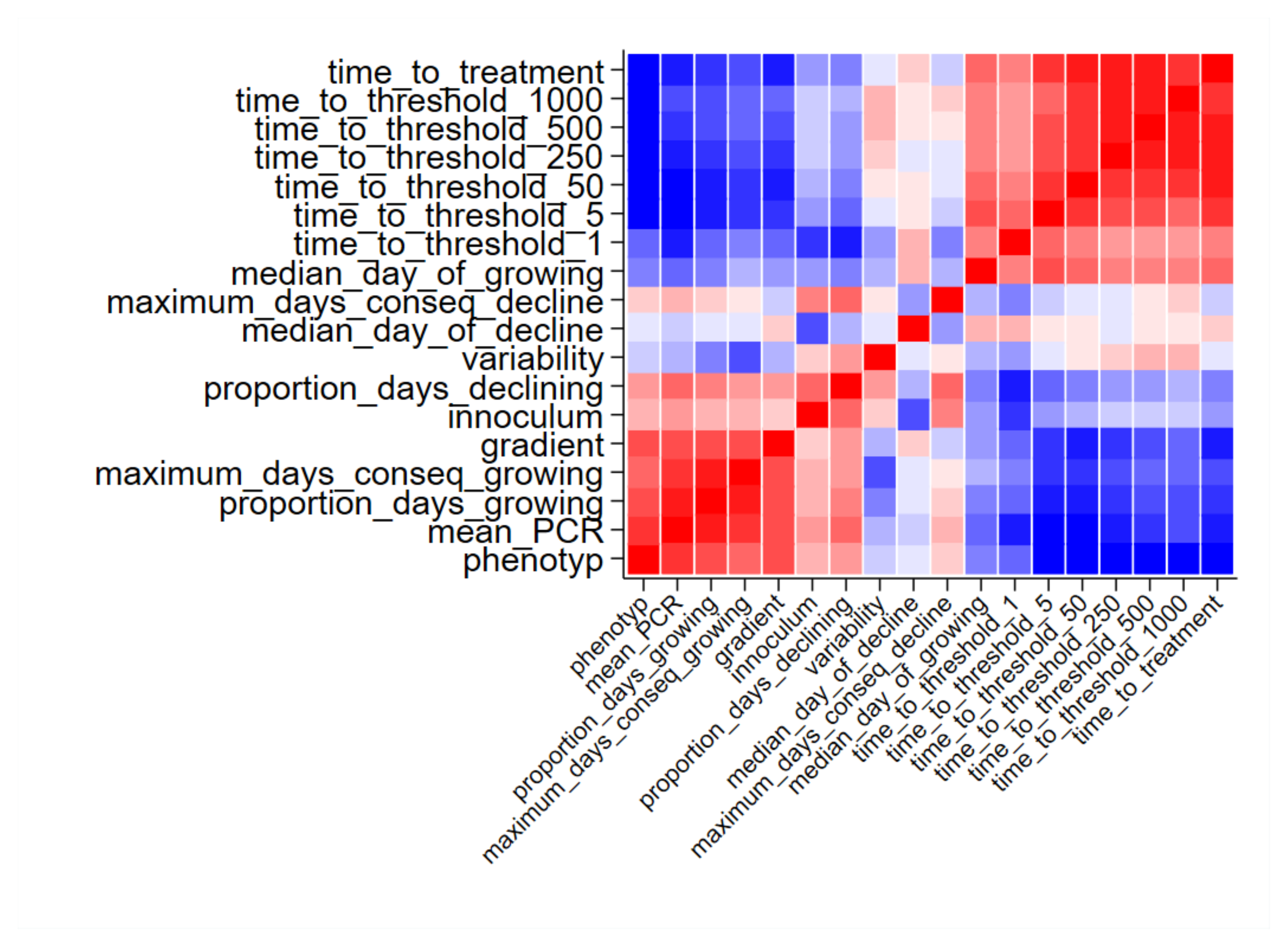

Supplement: Supplementary file 4 — Additional file 4: Figure S4. Spearman correlations of qPCR metrics. Correlation matrix of qPCR metrics showed collinearity for phenotype (treated vs untreated), time to thresholds (1000 parasites/ml; 500 parasites/ml; 250 parasites/ml; 50 parasites/ml; 5 parasites/ml; and 1 parasite/ml) or to diagnosis, and mean qPCR/gradient/days of growing (maximum days consecutively growing; median days of growth; proportion of days growing) or decline (maximum days consecutively decline; median days of decline; proportion of days declining), and other parasite growth metrics. Inoculum represents peak at days from days 8.5 to 10 post-infection; proportion of days with parasite growth represents analysis of smoothed data; proportion of days with parasite growth represents analysis of raw data; and variability represents the summed/average day to day increase or decrease. [file 12879_2022_7044_MOESM4_ESM.tif]
