# Supplementary material for: Controlled human malaria infection (CHMI) outcomes in Kenyan adults is associated with prior history of malaria exposure and anti-schizont antibody response
Source: BMC Infect Dis. 2022 Jan 24;22:86. doi: 10.1186/s12879-022-07044-8 (PMC8785382; doi:10.1186/s12879-022-07044-8)
Supplement: Supplementary file 5 — Additional file 5: Table S1. Sub-group analysis of qPCR outcome in relation to anti-schizont antibody responses. Table S2. Parametric analysis of qPCR parameters with anti-schizont antibody responses and location. [file 12879_2022_7044_MOESM5_ESM.docx]

**Additional file 5**

**Table S1: Sub-group analysis of qPCR outcome in relation to anti-schizont antibody responses**

|  |  | Anti-schizont antibody concentration (AU) | Proportion resident at high transmission |
| --- | --- | --- | --- |
| Outcome | N | Odds Ratio | |
| Treated non-febrile | 30 | 0.52 (0.20 to 0.85) | 0.56 (0.38 to 0.75) |
| Treated febrile | 26 | -0.1 (-0.3 to 0.09) | 0.31 (0.12 to 0.49) |
| Untreated PCR (+) – early/late | 19 | 0.85 (0.50 to 1.1) | 1 (1 to 1) |
| Untreated PCR (+) – early | 18 | 1.2 (1.0 to 1.4) | 1 (1 to 1) |
| Untreated PCR (+) – late | 16 | 0.76 (0.50 to 1.0) | 0.93 (0.81 to 1.0) |
| PCR (-) | 33 | 1.2 (0.95 to 1.5) | 0.93 (0.86 to 1.0) |

*p*=0.15 for the outcome categories on log likelihood ratio testing compared with the outcome categories for Table 1 in predicting schizont antibody concentrations. AU, antibody units. In parenthesis is the 95%CI. N number of volunteers in the analysis.

**Table S2: Parametric analysis of qPCR parameters with anti-schizont antibody responses and location**

| Parameter | Anti-schizont antibody concentration  (AU) | *p* value  anti-schizont antibody | Location | *p* value  Location |
| --- | --- | --- | --- | --- |
| Inoculum^a^ | 955 (776 to 1,202) | 0.74 | 0.53 (0.07 to 1) | **0.03** |
| Mean parasite density | 955 (646 to 1,445) | 0.86 | 0.7 (-0.08 to 1.47) | 0.08 |
| Proportion of days: parasite growth^b^ | 355 (794 to 1,259) | 0.11 | -0.42 (-2.7 to 1.93) | 0.73 |
| Proportion of days: parasite growth^c^ | 447 (63 to 3,236) | 0.42 | 1.66 (-2.2 to 5.57) | 0.4 |
| Proportion of days: declining parasite numbers | 1,349 (120 to 14,791) | 0.81 | 2.86 (-1.5 to 7.29) | 0.21 |
| Days of longest consecutive parasite decline | 933 (692 to 1,259) | 0.64 | 0.48 (-0.2 to 1.15) | 0.17 |
| Median point of days with parasite growth | 832 (708 to 955) | **0.02** | -0.23 (-0.61 to 0.15) | 0.24 |
| Median point of days with declining parasites | 1,000 (912 to 1,072) | 0.83 | -0.1 (-0.28 to 0.07) | 0.25 |
| Maximum days of consecutive decline | 933 (851 to 1,000) | **0.03** | -0.06 (-0.17 to 0.05) | 0.31 |
| Gradient | 1,288 (912 to 1,820) | 0.14 | 0.16 (-0.27 to 0.6) | 0.46 |
| Variability^d^ | 1,122 (1,023 to 1,230) | **0.01** | 0.13 (-0.04 to 0.29) | 0.13 |

^a^Peak at days considered are from days 8.5 to 10 post-infection; ^b^analysis of smoothed data; ^c^analysis of raw data; ^d^represents the summed/average day to day increase or decrease. AU, antibody units represented as median with 95%CI in parenthesis. Location is represented as proportion with 95%CI in parenthesis.
